# Thermodynamics of Competitive Molecular Channel Transport: Application to Artificial Nuclear Pores

**DOI:** 10.1371/journal.pone.0015160

**Published:** 2010-12-13

**Authors:** Wolfgang R. Bauer, Walter Nadler

**Affiliations:** 1 Department of Internal Medicine I, University Hospital of Würzburg, Würzburg, Germany; 2 Institute for Advanced Simulation (IAS), Juelich Supercomputing Centre (JSC), Forschungszentrum Juelich, Jülich, Germany; Dalhousie University, Canada

## Abstract

In an analytical model channel transport is analyzed as a function of key parameters, determining efficiency and selectivity of particle transport in a competitive molecular environment. These key parameters are the concentration of particles, solvent-channel exchange dynamics, as well as particle-in-channel- and interparticle interaction. These parameters are explicitly related to translocation dynamics and channel occupation probability. Slowing down the exchange dynamics at the channel ends, or elevating the particle concentration reduces the in-channel binding strength necessary to maintain maximum transport. Optimized in-channel interaction may even shift from binding to repulsion. A simple equation gives the interrelation of access dynamics and concentration at this transition point. The model is readily transferred to competitive transport of different species, each of them having their individual in-channel affinity. Combinations of channel affinities are determined which differentially favor selectivity of certain species on the cost of others. Selectivity for a species increases if its in-channel binding enhances the species' translocation probablity when compared to that of the other species. Selectivity increases particularly for a wide binding site, long channels, and fast access dynamics. Recent experiments on competitive transport of in-channel binding and inert molecules through artificial nuclear pores serve as a paradigm for our model. It explains qualitatively and quantitatively how binding molecules are favored for transport at the cost of the transport of inert molecules.

## Introduction

Understanding of molecular or particle transport through channels and pores is of paramount interest in many field, ranging from nanotechnology to life sciences [1]–[5]. In addition, such channel transport also serves as a paradigm for general linear transport processes like enzymatic catalysis with a 1-D reaction coordinate [6]. Optimal function of a channel in either a technical or biological setting often requires a high transport rate, which demands an adjustment of particle-channel- and interparticle interaction as well as particle concentration in the baths adjacent to the channel ends. The flow-facilitating role of a in-channel particle trapping, either by a binding site or an entropy trap, which prolongs the residence time and by this the translocation probability, has been recognized early [7]–[9]. When particles interact, however, this trapping hampers flow as it impedes access of other particles from the baths to the channel. This implies the existence of an optimum binding strength providing maximum flow [5], [10], [11], which depends on particle concentration, width and location, e.g. asymmetry, of the binding site [5], [12].

Despite of this previous work, many issues remain to be solved. How are the particle in-channel and interparticle interaction related to the occupation probability, i.e. a parameter observable in experiments? What is the exact mechanism responsible for an asymmetric binding site to favor transport selectively when located near the exit the flow is directed to? In which way is the optimum binding strength related to exchange dynamics at the channel ends? May also repulsive particle-channel interaction be favorable for transport? Which parameters determine the transition from a flow-facilitating binding site to a flow-facilitating repulsive interaction, and what is the mechanism behind? In a typical environment particles also compete with particles of other species for channel transport, each of them having their individual characteristics as in-channel affinity. The question arises how interspecies competition affects flow and how selectivity may be achieved e.g. by appropriate choice of in-channel interactions.

In this paper we will derive particle flow as a function of exchange dynamics and energetics at the channel ends, in-channel affinity, and interparticle interactions for single- and multi-species transport. The theory relates in-channel interaction directly to occupation probabilities of channel states, i.e. parameters accessible by experiments. A simple relation between exchange dynamics at the channel ends and particle concentration predicts whether a binding site or a repulsive force inside the channel facilitates transport. For the case that different species, each of them having its specific interaction profile, compete for channel transport, we analyze the influence of these interactions on flow of each species. Results are compared with recent experimental data [4] on transport through nuclear pores. Our model explains qualitatively and quantitatively the efficiency and selectivity of this transport process, which is of supreme importance e.g. for regulation of genomic activity.

## Methods

### The model

We consider particle transport through a channel connecting two baths, labeled as (A) and (B), with respective particle concentrations 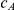 and 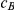. Particle motion in the channel is described as a 1-D diffusion process, and the dynamics of particle density 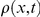 is given by the Smoluchowski Equation [13], [14],
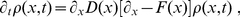
(1)


where 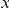 is the channel coordinate, giving the position of the molecule related to the channel, 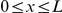, and 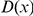 is the local diffusion coefficient, which is assumed to be constant, 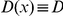. Particle-channel interaction is quantified by the force 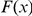 that can always be derived from a potential in the 1-D case, 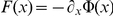. All energetic quantities are given in multiples of 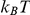, with 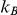 the Boltzmann constant.

**Figure 1:**
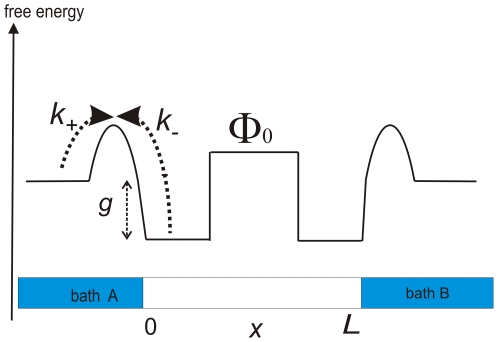
Free energy profile of a channel of length 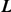 with reactive ends. The particle in-channel interaction 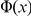 is here repulsive, with a barrier height 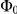. The reaction rates 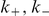 comprise the exchange dynamics at the channel ends, 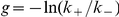 is the standard free energy of this reaction process.

The exchange rates of particles, entering or leaving either channel end are 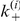 and 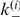, 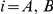 (Fig. 1). So the full transport process is described by the reaction-diffusion schematic
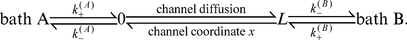
(2)


The free energy levels of the baths are assumed to be equivalent, which makes flow vanish for equal concentrations 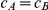. With
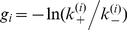
(3)


as the standard free energy of the reaction at the channel end 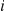, this condition is fulfilled when 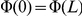 and 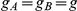. The more general condition for equivalent free energy levels of the baths, 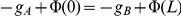, may always be transformed to the latter by appropriate gauging of 
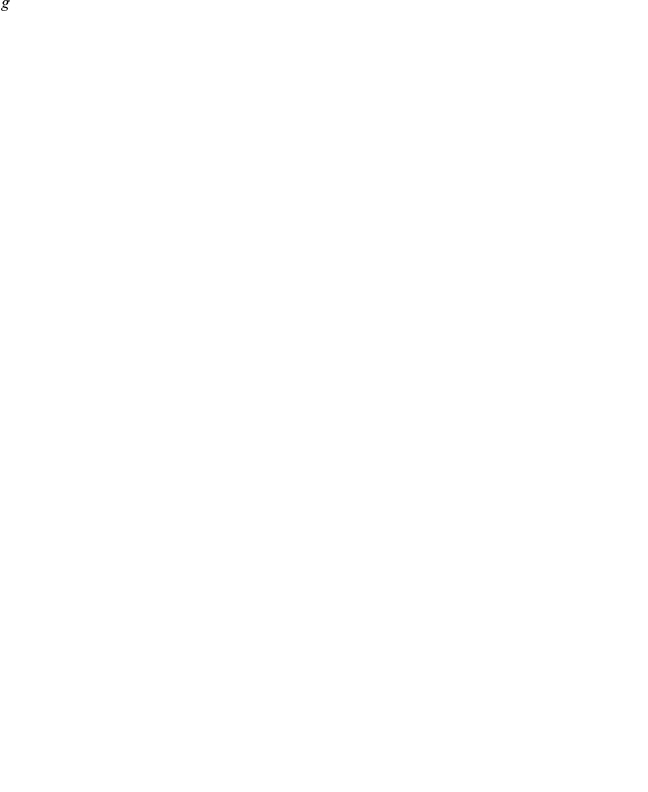
 and 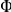 (see Appendix S1). Note that the rates 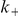 and 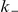 in Eq. 3 describe particle exchange between a three-dimensional space (bath) and one-dimensional space (channel) with corresponding 3D and 1D particle concentrations 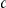 and 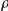 respectively. This is accomplished by assuming that the 3D particle concentrations at the channel entrances 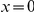 and 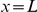 are practically constant perpendicularly to the channel (x) axis, i.e. 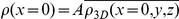, with 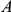 as the area of the channel opening. Hence, the equilibrium constant 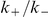, and consequently 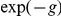, have units of an area, which we assume to be normalized by the channel opening area 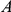.

## Results

### Interacting Particles of one Species

As a simple form of particle-particle-interaction it is assumed that a particle within the channel blocks access of particles from outside, a situation which is realistic especially for transport of large long molecules. Since this ansatz depends on a reduction of state space rather than on the neglect of correlations, we do not consider it a mean field type approximation. Now particles require an empty channel to enter some end, implying that the rate of particles entering the channel from the bath 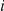 is not simply 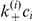, as it would be for non-interacting particles. Instead when we consider an ensemble of channels, particle transitions occur only in the fraction of empty channels. So when 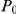 denotes the steady state probability that a channel is empty, we obtain that the ensemble averaged transitions per unit time from the bath to the channel end 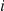 is 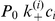. In the steady state particle density becomes stationary 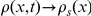 and flow 
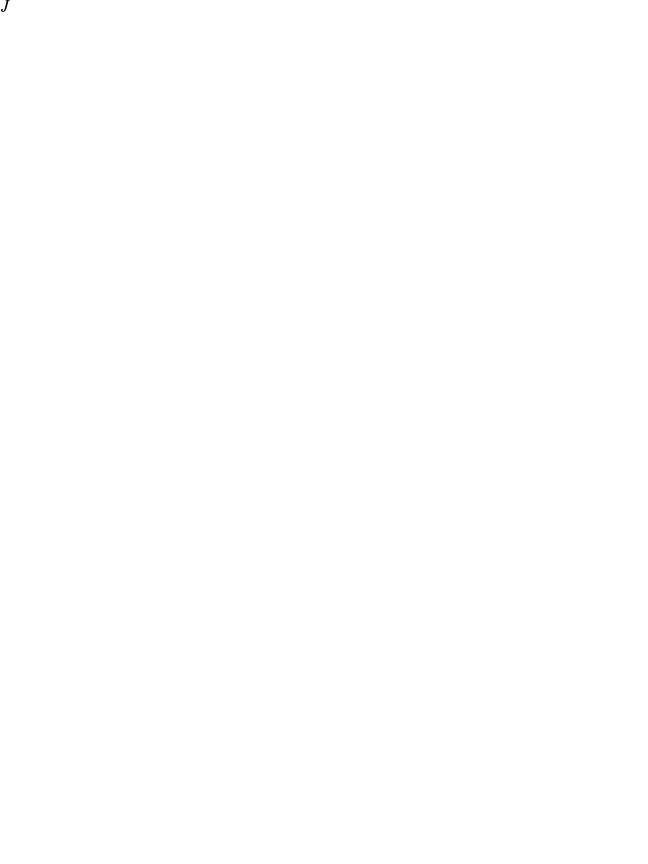
 is constant throughout, i.e. reactive fluxes at the channel end and diffusive flow are equivalent,
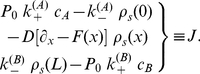
(4)


To solve the above equations it is useful to study first particle transport in the absence of particle-particle interaction, which is realized by setting 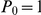. Here flow 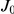 is derived as a macroscopic Fick's diffusion law (see Appendix S2 and Refs. [5], [9]),
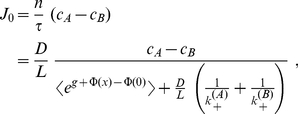
(5)


where
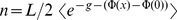
(6)


is the symmetrized specific particle number, which is a normalized measure of the number of particles occupying the channel, and 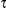 is the symmetrized first passage time, (see Appendix S2, Eqs. (S2-6)). The brackets denote the spatial average 
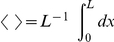
. Flow 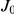 and its corresponding diffusive conductivity, 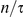, are directly related to the translocation probability (see Appendix S2), i.e. the conditional probability that a particle starting at one end of the channel is absorbed by the bath located oppositely 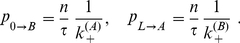
(7)


It is important to stress that flow 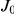, and hence translocation probabilities, increase with binding strength, and that they are invariant under permutations of the potential values 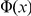, since they solely depend on the mean value of 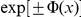. In particular any asymmetry of particle in-channel interactions is not reflected in flow, as long as particles are non-interacting.

The Eqs. (4) imply that switching from non-interacting to interacting particles is formally accomplished by replacing concentrations by their probability weighted values 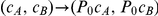 i.e. steady state flow derives formally as 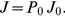
(8)


For the determination of 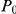, one applies conservation of probability, 
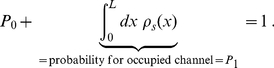
(9)


which gives (see Appendix S2)
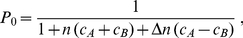
(10)


where 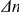 is the asymmetric counterpart of 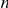, (see Appendix S2, Eqs. (S2-7)), i.e. it is a normalized measure of asymmetric occupation capacity which vanishes for symmetric interactions.

Equation (8) relates flow of interacting particles to flow of non-interacting ones, weighted by the probability 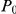 to find an empty channel. For low concentrations particle-particle interactions become negligible. This is reflected by 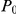 approaching unity (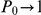), and flow approaching that of non-interacting particles 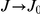. Since 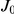 is invariant under permutations of the potential 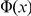 and interchange of exchange rates 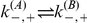, the Eqs. (8, 10) make clear that any asymmetry of flow is related purely to the asymmetry of channel blocking, i.e. to the asymmetry in the probability to find an open channel 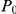. When asymmetry of flow is quantified by the difference of unidirectional flows at the same concentration 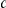, 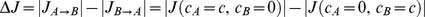, one obtains 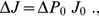
(11)


with 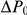 given in Eq. (S2-32) in Appendix S2. Asymmetry of flow depends either on asymmetry of the potential 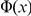, or on the difference between the exchange rates at the channel ends. We first discuss the case of equivalent exchange dynamics at both channel ends. Then a binding site located near that bath to which the flow is directed implies a higher probability to find the channel open than a binding site at the bath located oppositely, see also Appendix S2. Consequently, a binding site located in trans position of the concentration gradient implies a higher flow than in cis position. Next we consider that the potential 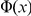 is symmetric, but the exchange rates at the channel ends differ. In this situation flow is lower when directed to the channel end with the lower exit rate, than in reverse direction. This is not a trivial observation! One might argue that identical free energies 
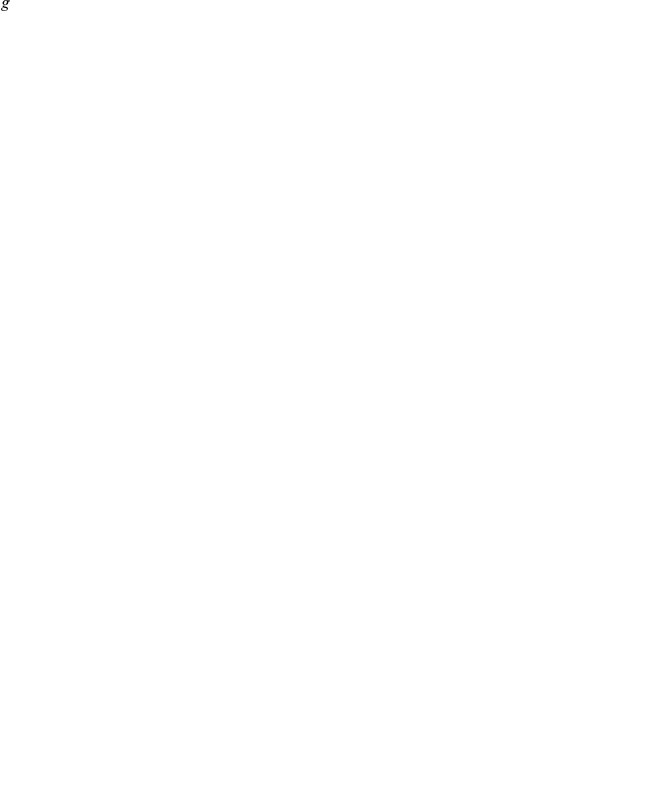
 at the channel ends imply that a lower exit rate is accompanied by a lower access rate as well. Therefore, reversing the concentration gradient should not alter flow. However, it turns out that the lower exit rate implies a higher occupation probability of the channel, which impedes flow. Asymmetry of the potential and of the exit rates may work synergistically, whenbinding site and low exit rate are located at opposite channel ends, or competitive, when both located at the same end.

#### Experimental Determination of Parameters

According to Eqs. (5,8,10) unidirectional flow as a function of concentration exhibits a saturation kinetics, equivalent to that obtained from the Langmuir or Michaelis-Menten Equation, in molecular adsorption or enzymatic kinetics, respectively. For facilitated carrier transport this kinetics has been suggested by Noble [15]. For channel transport it was observed in experiments on DNA transport through nanotubes [16]. The Langmuir or Michaelis-Menten constant, i.e. the concentration for which flow takes half the value of its saturation value, is
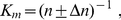
(12)


for unidirectional flow 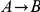, and 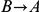, respectively. So kinetic experiments should provide the symmetrized and antisymmetrized specific particle numbers 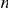, 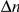 respectively. With channel length 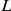, the free energy of particle channel interaction, 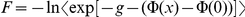, is then obtained from Eq. (6).

Alternatively, these parameters derive with Eq. (10) from ratios of occupation probabilities, obtained for unidirectional transport at identical concentrations
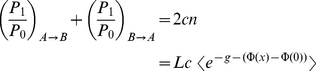
(13)

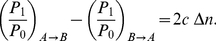
(14)


The last equations have a strong impact: Ratios of occupation probabilities are equivalent to ratios of corresponding lifetimes of channel states (see Appendix S2). The latter may be obtained experimentally by conductance measurements. From a more theoretical point of view it is of interest that symmetrized ratios of occupation probabilities, determined in the steady state, i.e. under non-equilibrium conditions, are equivalent to the Boltzmann factor corresponding to the free energy of the particle channel interaction, which, as well known, is equivalent with the ratio of the equilibrium occupation probabilities.

#### Optimal Transport

To determine the in-channel interaction for maximum transport we restrict ourselves to interactions corresponding either to wells or barriers and do not consider potentials oscillating around zero. The probability 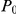 to find an empty channel increases monotonically with increasing 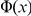, from zero for strong binding, blocking all channels, and approaches asymptotically some value below unity. Concomitantly, 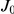 decreases from some value below the finite upper threshold determined by 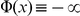 in Eq. (5), and reaches zero for infinitely high barriers. So, flow for interacting particles, 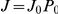, vanishes for strong binding as well as for high barriers, which implies the existence of some maximum at an intermediate interaction 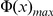.

**Figure 2:**
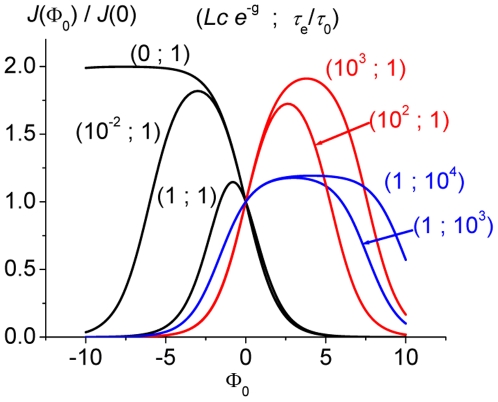
Flow through a channel with symmetric rectangular shaped potential (relative width 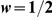, depth or height 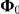, normalized by flow at vanishing interaction. Different chemical activities and exchange dynamics at channel ends are studied 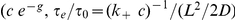. Maximum of flow shifts toward weaker binding strength, and may even appear at repulsive interactions (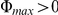), when chemical activity increases (red lines), or exchange dynamics slows down (blue lines).

**Figure 3:**
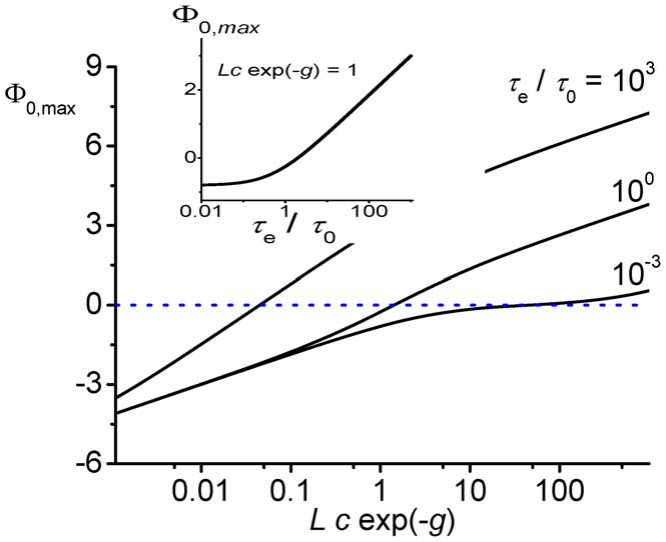
Channel-particle interaction at maximum flow 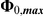, as a function of the activity 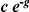, and exchange dynamics 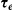 (insert). Other parameters are as in Fig. 2.

The Eqs. (5,10) imply that 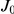, 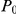, and, hence, 
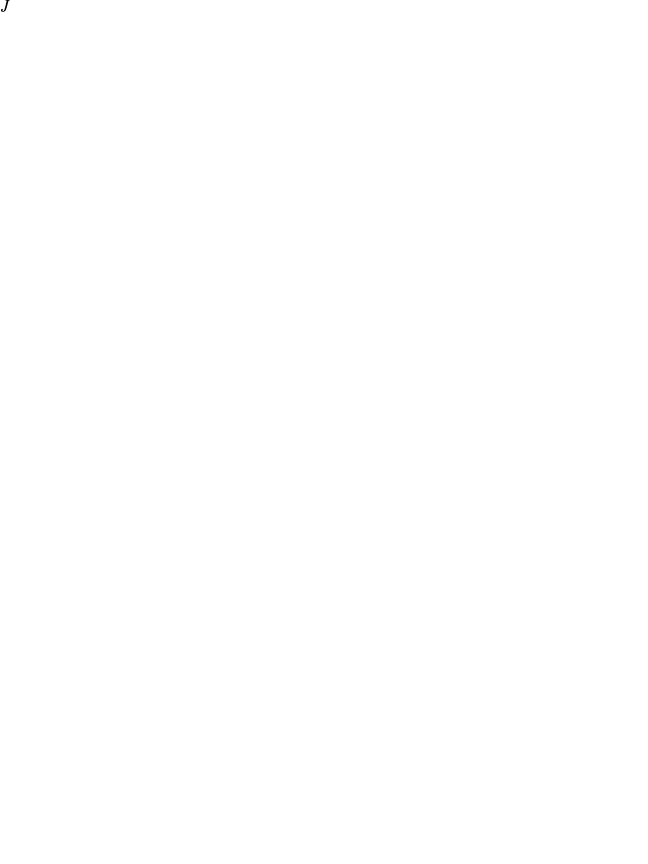
 remain invariant under renormalization of the interaction 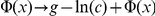. This property has interesting consequences for the value of 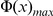. With increasing activity 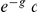, 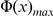 must increase to compensate for channel blocking and may become repulsive (positive) above some threshold, see (Figs. 2, 3). The value of 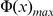 further depends on the exchange dynamics at the channel ends, i.e. on particle mobility and energetic or entropic barriers in this region (Fig. 1).

To analyze this in more detail we next study the variation of flow, 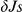, at the transition from attractive to repulsive in-channel interaction, 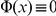, for a small positive variation of in-channel interaction 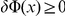. A negative 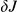 implies the existence of maximum of flow at an attractive interaction, 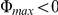. Vice versa, a positive 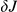 implies some maximum for a repulsive interaction, 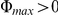. Since 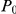 is independent of the exchange dynamics, 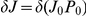, is influenced by it only via 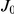, Eq. (5). For sufficiently slow dynamics, i.e. when 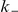 is very small, exchange at the channel ends becomes the time limiting step. In that case 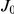 scales at 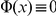 with 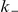, and its variation 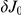 with 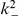, i.e. the latter becomes negligibly small. So the variation of 
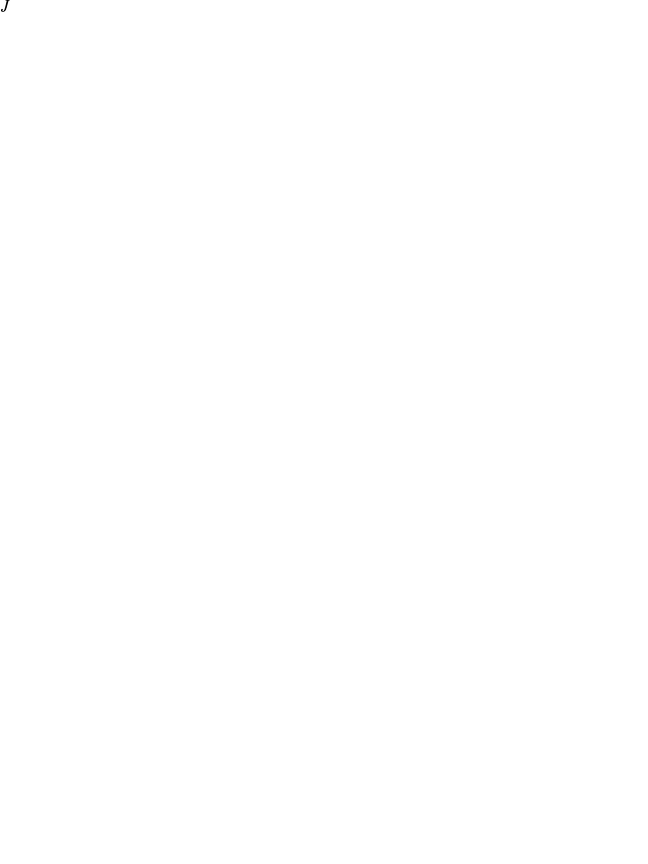
 fulfills
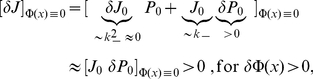
(15)


where we exploited the monotony of the function 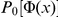. Hence, in the limit of slow exchange dynamics at the channel ends the switching on of a small repulsive interaction, 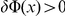, leads to an increase in flow, an effect that is somewhat counterintuitive at first. However, the reason for that effect is that the channel blocking is reduced to such an extent (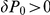) that it dominates the flow impeding effect of the repulsive interaction on translocation probability 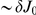.

Explicit evaluation of the variation, Eq. (15), by its functional derivative then provides the relation between channel end activity and exchange dynamics determining the value of 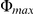,
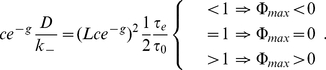
(16)


Here we introduced the mean time the channel stays empty 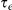, related to the time scale of mobility within the channel, as given by the mean first passage time 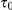 of a particle freely diffusing a distance 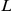,
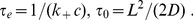
(17)


The relation determining the exit rates at which the optimal potential switches from attractive to repulsive, resulting from Eq. (16), is given by
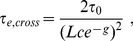
(18)


with 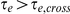 leading to a repulsive optimal barrier, 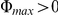.

As a paradigm we study a symmetric rectangular shaped potential 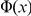 of relative width 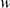,
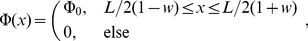
(19)


which acts as a well, for 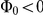, or barrier when 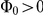. The interaction determining maximum transport is obtained from the condition 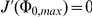 for 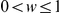 as
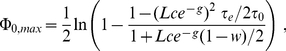
(20)


with corresponding maximum flow
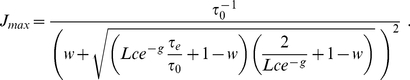
(21)


Increasing activity of particles 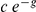, or slowing down exchange dynamics at channel ends shift 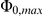 toward weaker binding (Figs. 2, 3). One can easily verify that the threshold determining the transition from attractive to repulsive optimal interactions in the rectangular well in Eq. (20) is given by the general results of the variational approach, Eqs. (16,18).

### Competition of Different Species: Comparison with Experimental Results in Nuclear Pore Transport

**Figure 4:**
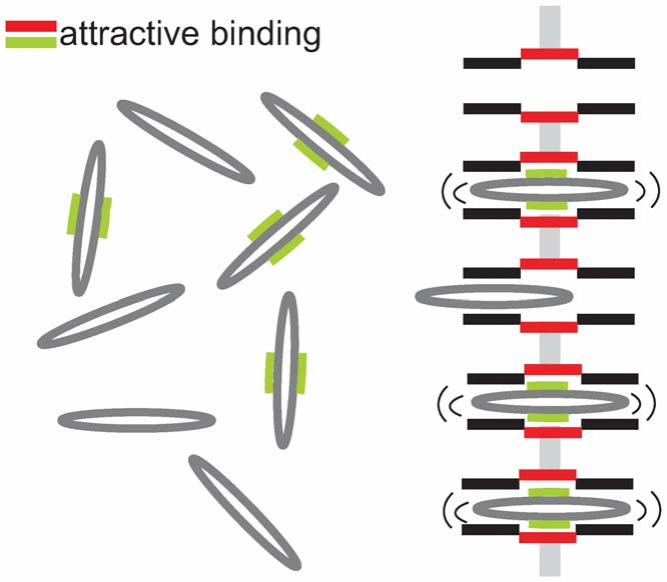
Two species competing for transport through channels. One has the capability of in-channel binding (green-red binding sites), the other is inert. The binding species dominates in-channel sojourn, and by this increases translocation probabilit. Hence, transport of the binding species is increased on the cost of the inert species, the channel access of which is hampered.



In this section we consider different species of molecules, labeled by the superscript 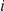, with concentrations 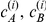 in respective baths, which compete against each other for channel transport (Fig. 4). Each of the species may have its specific channel affinity. We assume the intra- and interspecies interaction of molecules as above, i.e. a channel occupied by one molecule blocks channel access of any other molecule. This implies that steady state flow 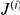 at the channel ends is proportional to the probability 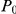 to find the channel non-occupied as given by Eqs. (4). However, the probability 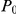 now depends on the concentration and on the binding properties of all species. Based on the conservation of probability one derives for 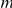 different species, similarly to Eq. (10) (see Appendix S2, Eq. (S2-20))

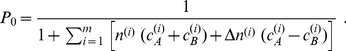
(22)Flow of the 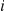-th species is the product of the probability 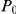 times the flow in the absence of any intra- and interspecies interaction of molecules 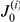, i.e.
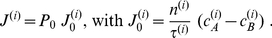
(23)


Equation (22) states that all species contribute to the reduction of probability to find an empty channel proportional to their in-channel affinity and concentration. This effect uniformly hampers flow in all species, see Eq. (23). Selectivity results solely from the effects on the translocation probability of the particular species, which is proportional to 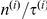, see Eq. (7). This implies that the ratio of flows of two species 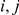 is independent of interspecies interactions, since 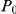 cancels,
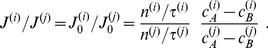
(24)


Note that this ratio also does not depend on permutations of the respective interactions.

We assume in the following that the species are similar in their exchange dynamics at channel ends (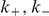), and in their diffusion properties (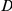). The conductivity of unidirectional transport, 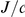, of a binding species 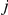 always exceeds transport of a non-binding species 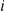, since (see Eq. (5))
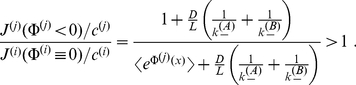
(25)


Note that we gauged for simplicity the interaction at the channel ends to zero, i.e. 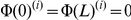 for all species. This is not a restriction, as finite variations of the interaction at singular points do not affect the diffusive process.

Increasing the binding strength of a particular species 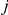, i.e. 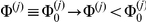, implies that the probability to find the channel empty decreases. This has the effect that flow of all other species 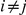 decreases proportionally as
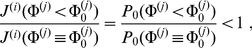
(26)


see also Fig. 4. The flow ratio of the species with increased binding, 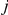, behaves more complicatedly. It changes to
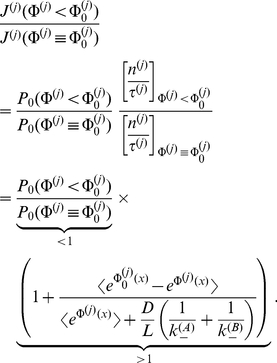
(27)


**Figure 5:**
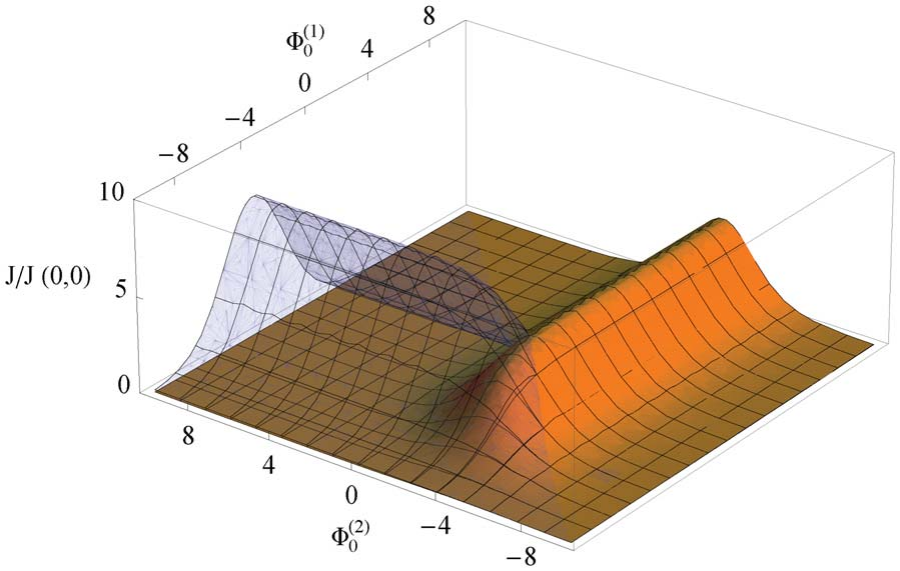
Unidirectional flows of two competing species, transparent blue, label (1), and yellow, label(2), as a function of respective particle-channel interactions 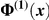 and 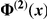. Flows are normalized to that in the absence of particle-channel interactions. A symmetric rectangular shaped potential with relative width 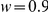, and potential height/depth 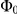 is assumed. The activities of species were chosen as 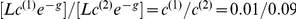, i.e. a 1∶9 mixture. A very fast access is considered, 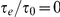.

As was the case for single species transport, the increase of binding strength has different effects on the occupation probability and on the translocation probability, 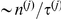. While the occupation probability decreases, the translocation probability increases. Following the same arguments as for single species transport, varying the binding strength from infinitely high values to infinitely low values (corresponding to completely repulsive interaction), lets the flow vary from zero through some maximum value to zero again. Figure 5 illustrates this behavior.

To summarize, the flow of a species decreases monotonically with increasing binding strength of its competitor. If the binding strength for maximal flow of this competitor is sufficiently strong, i.e. 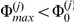, then the flow of the latter increases. In this case facilitated transport of the binding species on cost of the other species is possible.

**Figure 6:**
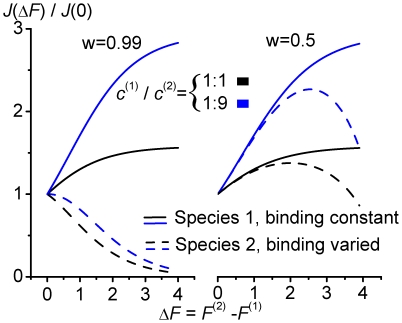
Unidirectional flows of two competing species, labeled (1) and (2), with symmetric rectangular shaped particle channel interactions. The free energy of binding 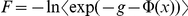 is held constant for species one at 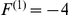, whereas that of species two is varied. Two relative widths of particle channel interaction 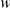, and two different mixtures of species are considered 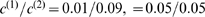. Access of the two species was assumed to be very fast, 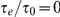.



To analyze selectivity more closely, we investigate unidirectional flow of two species of same particle concentration and initially the same symmetric particle channel interaction 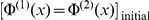 and reduce the binding strength of the second one, i.e. 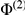 increases (Fig. 6). With the free energy of particle in-channel interaction, 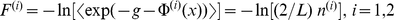, see Eq. (6), this implies 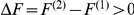, and 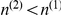. So, one obtains for the flow of the species, when normalized to flow for initially equivalent interaction, 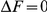 (see Eqs. (22–23)),

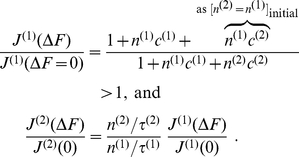
(28)Weakening the binding strength of the second species reduces the probability that the channel is blocked, and by this, facilitates transport of the first.

The effect on the second species is more complex. A reduction of its binding strength reduces its translocation probability, 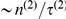, see Eq. (5,7). However the flow hampering effect of blocking is reduced as well. Following the same arguments as in the previous section there exists a maximum of 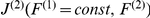 at some value 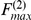, when 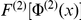 is varied from infinitely high to low (repulsive) binding strengths. Hence, the behavior of the flow 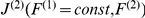 for increasing values of 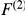 depends on the location of 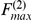. Flow of the second species decreases with decreasing binding strength when 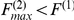, see the left panel of Fig. 6, i.e. small variations of the binding strength have a strong effect on selectivity. Vice versa, when 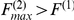, a decreasing binding strength makes flow first run through some maximum, before it decreases (right panel Fig. 6). Hence, larger variations of binding strength are necessary to achieve selectivity. The first scenario,in which small variations of binding strength of the second species resulted in transport selectivity, demands that maximal flow of this species occurs at a sufficient strong binding strength, so that 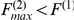. This condition is best fulfilled when the width of the binding region is large, and channel-solvent access dynamics is fast, see Eq. (20).

**Figure 7:**
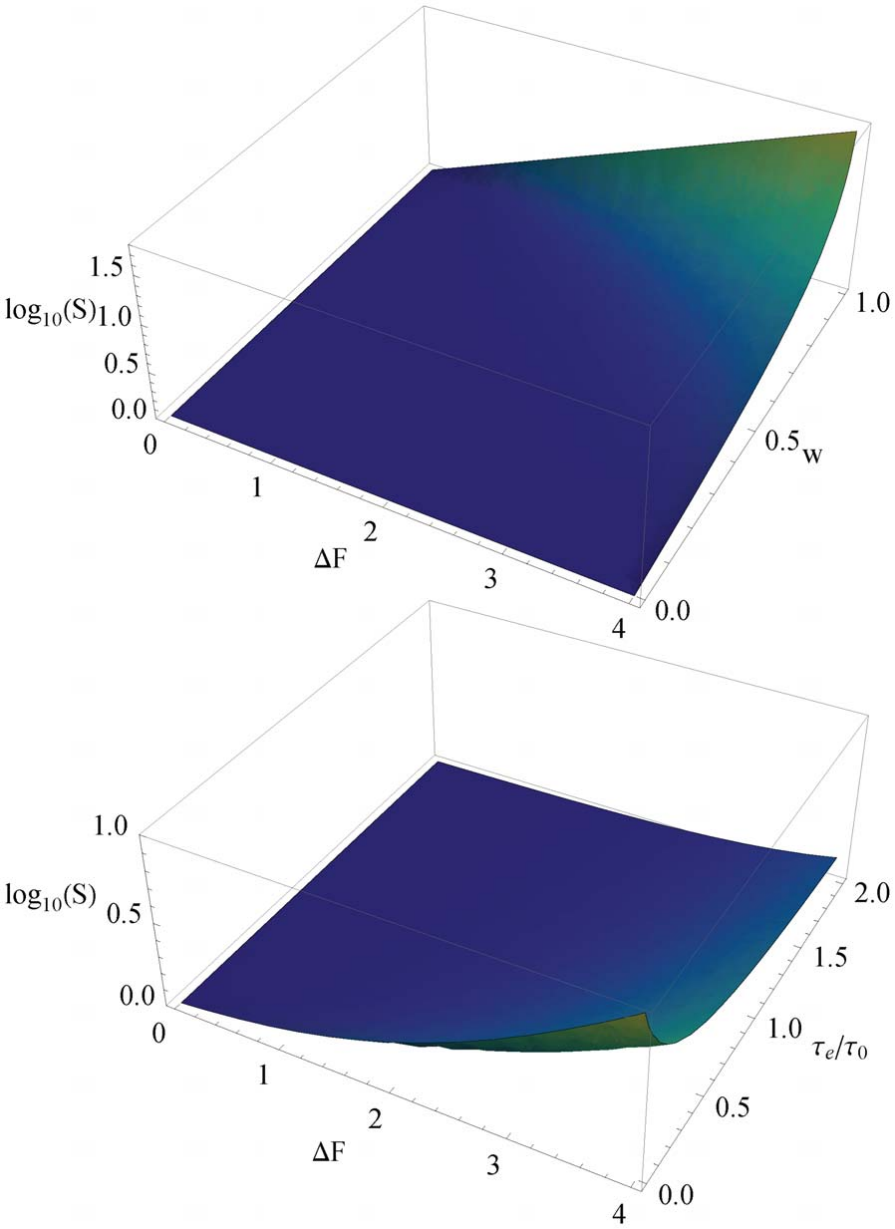
Selectivity, defined as the ratio of relative flows for two species 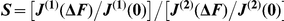, as a function of the difference of free binding energy 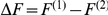, and relative width of the rectangular potential is shown in the above panel. The access dynamics is here assumed to be very fast 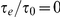. In the panel below 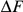 and the ratio of time scales of access and transport dynamics, 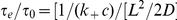, are varied. The relative width was fixed to 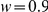).

When we quantify selectivity as the ratio of relative flows of the two species we get
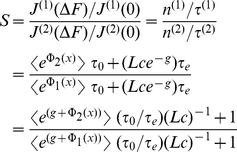
(29)


i.e. it is identical to the ratio of the translocation probabilities, see Eqs. (5–7). Figure 7 demonstrates that this selectivity increases with the width of the binding site, as does the translocation probability for a binding site. Selectivity works better the faster the access dynamics is in relation to the time scale of channel crossing, measured by the corresponding time scales 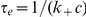 and 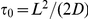, respectively. In other words, selectivity works better the more the species differ in their binding strength, the longer the channel is, the slower diffusion is within, and the faster the particles enter the channel from outside.

**Figure 8:**
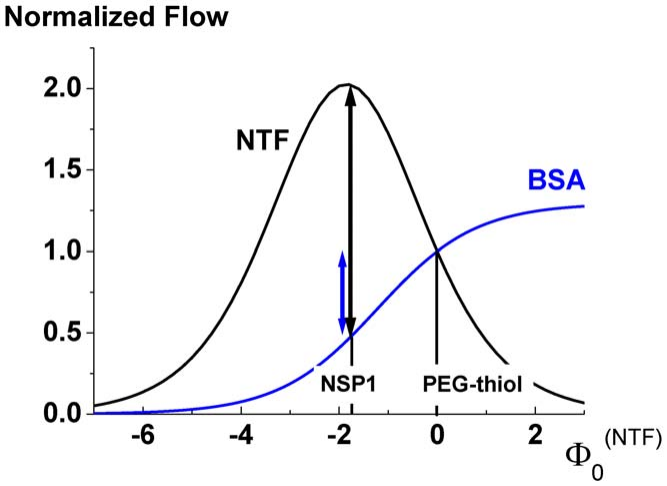
Competitive transport of bovine serum albumin (BSA, blue line) and nuclear transport factor (NTF, black line) through a nuclear pore as a function of the NTF in-channel interaction. The interaction potential is assumed to have a symmetric rectangular shape, with height/depth 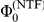 and relative width 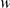. Fluxes are normalized to fluxes in a pore functionalized with PEG-thiol, in which NTF interaction vanishes, 
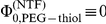
. BSA is inert for in-channel interaction of any functionalized pore, i.e. 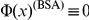. Experiments revealed that BSA flow through a pore functionalized with NSP1 is about 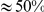 (blue arrow) of that through a PEG-thiol pore, whereas NFT flow is about 4 times that of BSA flow (black arrow) [4]. This determines the relative width as 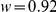, and binding energy 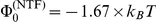, see the main text. Concentrations were 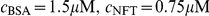 [4], and translated into corresponding activates 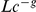, see Appendix S3.

This theory explains experimental results on selectivity and competition in artificial nanopores mimicking the nuclear pore complex [4]. These pores contain nucleoporins which transiently bind to transport factors plus cargo, and by this control flow of the latter through the nuclear envelope. The authors investigated competitive transport of the human nuclear transport factor 2-gluthatione S-transferase, NTF2-GST (NTF), and of bovine serum albumin (BSA), which is similar in size and diffusion properties to NTF. The pores were functionalized either with nucleoporins (NSP1) or PEG-thiol, which are comparable in size and polymer properties. However, the NTF binds solely to the NSP1 pore, but not to the PEG-thiol one. The inert BSA binds to neither of the functionalized pores. After replacement of the PEG-thiol by the NTF binding NSP1 pore, BSA flux decreased, whereas that of the competing NTF increased. This is shown in Fig. 8, where the binding strength of the NTF is varied. An increasing binding strength (decrease of 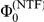) monotonously decreases the flow of the inert molecule (BSA), whereas that of the binding NTF runs through some maximum. An increasing binding strength of NTF increases its translocation probability, 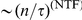. Since exchange dynamics at the channel ends is sufficiently fast (see Appendix S3), this effect of NTF binding dominates that of reducing 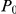, implying the existence of some optimal binding strength, 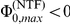, at which maximum NTF flux occurs. Conversely, the translocation probability of the inert BSA, 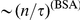, is not affected, and BSA flow is reduced due to the NTF-binding related decrease of 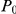, Eq. (26).

Our model does not only describe qualitatively the experiments, but also provides some quantitative insights into the binding energetics. From the data of Jovanovic-Talisman et al. one can determine the ratio of diffusive conductivities of NTF and the inert BSA, Eq. (25), for a pore functionalized with the nucleoporin NSP1, i.e. 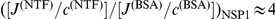 (see Appendix S3). The access of transport factors to the channel ends, and hence exchange dynamics here, may be estimated to be very fast when compared to transport inside the channel (see Appendix S3). This implies that the ratio of conductivities of binding NTF (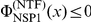) and non-binding BSA (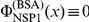) in Eq. (25) simplifies to
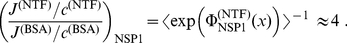
(30)


The observed value of approximately 50% for the reduction of flow of the inert BSA molecule competing with NTF when switching from the non-binding PEG-thiol pore to the NTF binding NSP1 pore determines with Eq. (26) the ratios of probabilities,
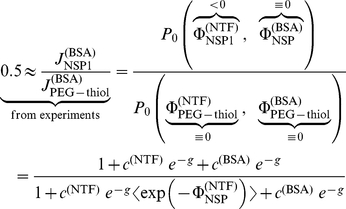
(31)


where we assumed a symmetric channel. The above equation reveals after insertion of activities and structural data (see Appendix S3), the free energy 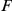 of in-channel interaction of NTF is
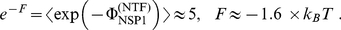
(32)


The average of the Boltzmann factor and its inverse in Eqs. (30,32), determined from experimental data, correctly reflect the Cauchy-Schwartz inequality
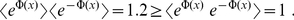
(33)


This product is unity if, and only if the interaction is constant throughout the channel, i.e. 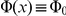. Hence, a product close to one, as it is the case above, implies that 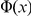 is still approximately constant over a significant domain of the channel. In fact, approximating particle channel interaction by a rectangular potential (Eq. (19)), which selfconsistently reproduces the average Boltzmann factor and its inverse (Eqs. (30,32)), provides a relative width of 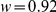, i.e. close to unity, and a binding energy of 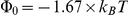, which is close to the free energy of in-channel interaction.

## Discussion

The model presented here allows us to analyze how interparticle interactions, particle in-channel energetics, as well as exchange dynamics and energetics at the interface of channel and solvent affect particle transport. Exchange at the interface was simplified by a two-side exchange process between solvent and channel end, where the corresponding rates comprise the dynamics of energetic or entropic barrier crossing. Inside the channel, diffusive particle dynamics is subject to forces which derive from an in-channel potential. Our model may also be extended to include entropic forces/barriers within the channel as presented by Reguera and Rubi [17]; however the detailed analysis would be beyond the scope of this article.

Interparticle interaction was approximated by the assumption that a molecule inside the channel completely blocks the access of others, i.e. interactions of several particles within the channel were excluded. This single occupancy condition has been described very early in literature for discrete and continuous models in the limit of fast solvent-channel exchange [18], [19] and is meanwhile often applied in models of channel transport [5], [10]–[12], [20], [21]. Discrete models which considered multiple occupancies for single file [22]–[24], or non-single file transport [25]–[27] were suggested in the past. However, analytical solutions for these models require that the discrete model is restricted to very few sites, or that the interaction force inside the channel takes a simple form, e.g. it is constant or even vanishes. For the latter case, which in the continuum limit corresponds to a constant potential, the effect of interparticle interaction on flow cancels on average for single species transport within the channel, but is present at the channel ends [26]. As long as single-file transport is a valid approximation, our model can be extended to include the interaction of several particles within the channel by adapting the condition of conserved probability in Eq. (9) for the maximum number of particles occupying the channel.

We derived flow explicitly in terms of occupation probabilities, free energy of particle in-channel binding and exchange dynamics. This allowed us to determine the free energy of particle-channel interaction, i.e. a measure of binding strength, from key parameters of the Michaelis-Menten kinetics, Eq. (12, as well as to determine the occupation probabilities, Eq. (14). Both quantities are accessible by experiments.

Flow with interparticle interaction could be factorized into a term 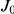 that describes only non-interacting particles, and into the probability 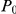 to find a non-occupied channel. Since 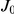 is proportional to the translocation probability and independent of the actual form of the in-channel interaction 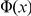, the only influence of the actual form of that interaction potential on flow is through its effect on 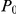. An important application of this results is the asymmetry of transport: When the direction of the concentration gradient is reversed, flow is higher when the binding site is located near the channel end of lower concentration. This result was derived in the past by us [5] and others [12] from respective models. However, it is now clear that it this result is related mainly to the asymmetry in the occupation probability.

We analyzed in detail the binding strength for maximal flow. In the past, scenarios had been discussed in which attractive interactions favored transport [5], [10], [12], [26], [28], explaining experiments e.g. for DNA [1] and nuclear pore transport [4]. Here we demonstrated that for high chemical activity of particles, or a slow exchange dynamics at channel ends, maximal transport can occur also for repulsive interactions. The effect of high activity 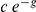 was also reported by Kolomeisky [20]. The effect of slow exchange dynamics, which, for example, may be due to energetic or entropic barriers a molecule has to pass at the channel entrance, is new, and required a kinetic explanation. The flow enhancing effect of repulsive interactions was not observed in our previous work [5], where exchange dynamics at the channel ends was assumed to be much faster than the first passage time to pass the channel. In that limit only attractive interactions can optimize transport.

We could extend our model straightforwardly to describe particles of different competing species, each having its own specific channel affinity. Binding favors flow of one species, if its effect on increasing the translocation probability exceeds its flow hampering effect due to increased channel blocking.

Note that only the latter effect, increased channel-blocking, affects the non-binding species, i.e. its flow is reduced when compared to vanishing binding. So binding of a species may enhance its flow on cost of the non-binding species.

We demonstrated for two species, both having initially equivalent particle-channel interaction, that a reduction of the binding strength of one species leads to an increased flow of the other. Flow of the species with reduced binding strength exhibits a more complex behavior. If the binding strength for maximal flow of this species is lower than the initially equivalent binding strength, flow for this species goes through a maximum before it declines with decreasing binding, see Fig. 5. Otherwise, flow of the species with reduced binding strength declines promptly and continuously, which is more favorable to achieve selectivity, see Fig. 6. This behavior of the flow was also observed in simulations of a multi-occupancy model for two competing species in [25], [27]. Interestingly, that multi-occupancy model revealed some moderate cross-dependence of the translocation probability of one species on the binding strength of the other. So the translocation probability of the species with conserved binding strength went through some maximum while reducing the binding strength of its competitor [27]. This feature is related to the fact, that the channel allowed multi-occupancy. A reduction of binding strength implies less particles of this species in the channel, i.e intra-channel interactions with the competing species, having conserved binding strength, decrease, which results in an increased translocation probability. In the single occupancy model, i.e. the model described here, the translocated particle blocks the channel during the whole process. So translocation of one species is independent of interparticle interactions, i.e. in particular independent of interactions with the other species. So, as discussed above, cross interactions derive solely from cross dependencies of occupation probabilities.

Our model also explains the experimental data for competitive transport of nuclear transport factors through artificial nuclear pores described in Ref. [4]. There flow of two competing species through pores was investigated. The two transported species were inert or potentially binding, respectively, while the pores were functionalized either with binding or inert sites. As derived from our model, the flow of the binding (non-binding) species within the pore functionalized with binding sites increased (decreased) when related to that of the inert pore. These ratios of flow also allowed a quantitative estimation of the strength and extent of binding.

## Supporting Information

Appendix S1Herein the interdependence of the particle-channel interaction potential 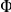 at the channel ends and the exit rates 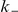 is analyzed, which allows appropriate gauging of both.(PDF)

Appendix S2 This Appendix derives in detail the dependence of channel flow on first passage time and channel occupation number and probability. These parameters are related to the translocation probability and the lifetime of channel states. In this context the effect of asymmetry of in-channel binding site on flow is derived.
(PDF)

Appendix S3The access dynamics of the nuclear transport factor (NTF) from outside to the nuclear pore is estimated. Furthermore the diffusive conductivities of bovine serum albumin (BSA) and nuclear transport factor (NTF) through the nuclear pores and their activities 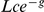 are computed from experimental data.(PDF)
