# Supplementary material for: Thermodynamics of Competitive Molecular Channel Transport: Application to Artificial Nuclear Pores
Source: PLoS One. 2010 Dec 13;5(12):e15160. doi: 10.1371/journal.pone.0015160 (PMC3001458; doi:10.1371/journal.pone.0015160)
Supplement: Appendix S1 — Herein the interdependence of the particle-channel interaction potential at the channel ends and the exit rates is analyzed, which allows appropriate gauging of both. (PDF) [file pone.0015160.s001.pdf]

Appendix S1

Gauging of potential Φ and exit rate k_- at the channel ends We will show that flow dynamics within the channel remains invariant under appropriate gauging of the potential $\Phi(x)$ and the exit rates k_- at the channel ends, $x = 0,\ x = L$. It is sufficient to consider this at the channel end $x = L$, where both shall transform according to

$$\Phi(x) \ \rightarrow\ \tilde{\Phi}(x) = \begin{cases} \Phi(x), & 0 \leq x < L, \\ \tilde{\Phi}(L), & x = L, \end{cases}$$

$$k_-^{(B)} \ \rightarrow\ \tilde{k}_-^{(B)} = e^{\tilde{\Phi}(L)-\Phi(L)}\ k_-^{(B)}\ . \tag{S1-1}$$

This transformation conserves the free energy level of bath B, $-g_B + \Phi(L)$, since the standard free energy of channel exchange transforms

$$g_B \rightarrow \tilde{g}_B = -\ln(k_+^{(B)}/\tilde{k}_-^{(B)}) = \tilde{\Phi}(L) - \Phi(L) + g_B\ , \tag{S1-2}$$

i.e. $-g_B + \Phi(L) = -\tilde{g}_B + \tilde{\Phi}(L)$. Note that the direction of channel exchange is defined from the bath into the channel, i.e. to obtain the free energy level of the bath, $-g$ has to be added to the potential Φ at the channel end.

Within the channel and its ends dynamics of particle density $\rho(x,t)$ is determined from the Smoluchowski Equation respecting the boundary conditions for flow density j at the channel ends, i.e.

$$\partial_t \rho(x,t) \ =\ \partial_x \underbrace{D(x)[e^{-\Phi(x)}\partial_x e^{\Phi(x)}]\ \rho(x,t)}_{=\,-\text{flow density}=-j(x,t)}, \tag{S1-3}$$

$$\begin{aligned} j(0) &= -k_-^{(A)}\ \rho(0,t) + k_+^{(A)} c_1 \\ j(L) &= k_-^{(B)}\ \rho(L,t) - k_+^{(B)} c_2\ , \end{aligned} \tag{S1-4}$$

where the Smoluchowski Equation was written in terms of potentials. Note that solutions of Eq. (S1-3) must provide a continuous flow density over the whole channel length, i.e. in particular do the boundary conditions claim

$$\lim_{x\rightarrow 0, x>0} j(x,t) = j(0,t), \text{ and } \lim_{x\rightarrow L, x<L} j(x,t) = j(L,t)\ . \tag{S1-5}$$

The transformed particle density

$$\rho(x,t) \rightarrow \tilde{\rho}(x,t) = \begin{cases} \rho(x,t), & 0 \leq x < L \\ e^{-(\tilde{\Phi}(L)-\Phi(L))}\rho(L,t), & x = L \end{cases} \tag{S1-6}$$

obviously solves Eq. (S1-3) for the potential $\tilde{\Phi}(x)$ for $0 \leq x < L$. This implies the equivalence of flow densities of the original and transformed process for $0 \leq x < L$

$$\tilde{j}(x,t) = j(x,t)\ . \tag{S1-7}$$

At the boundary $x = L$ the flow density of the transformed process is according to Eqs. (S1-1,S1-4)

$$\tilde{j}(L,t) = \tilde{k}_-^{(B)}\tilde{\rho}(x,t) - k_+^{(B)} c_2 = j(L,t)\ , \tag{S1-8}$$

i.e. the equivalence of flow density of the original and transformed process holds over the whole channel. Vice versa does this imply that $\tilde{\rho}(x,t)$ solves the Smoluchowski Eq. (S1-3) with potential $\tilde{\Phi}$ and the boundary condition (S1-8). In summary gauging of the potential and exit rate at the boundaries according to Eqs. (S1-1) conserves flow dynamics over the whole channel. Dynamics of particle density is not affected as well, except at the boundary, where the potential shift changes particle density by multiplying with the corresponding Boltzmann factor. However this finite singular change of particle density has no relevance for occupation probabilities over a non-vanishing spatial extent.

In particular: when baths are on the same free energy level, i.e. $-g_A + \Phi(0) = -g_B + \Phi(L)$, one may gauge to equivalent potentials and standard free energies at the channel ends, namely by setting $\tilde{\Phi}(L) = \Phi(0)$ which implies with Eq. (S1-2), $\tilde{g}_B = \Phi(0) - \Phi(L) + g_B = g_A$.
