# Supplementary material for: Thermodynamics of Competitive Molecular Channel Transport: Application to Artificial Nuclear Pores
Source: PLoS One. 2010 Dec 13;5(12):e15160. doi: 10.1371/journal.pone.0015160 (PMC3001458; doi:10.1371/journal.pone.0015160)
Supplement: Appendix S2 — This Appendix derives in detail the dependence of channel flow on first passage time and channel occupation number and probability. These parameters are related to the translocation probability and the lifetime of channel states. In this context the effect of asymmetry of in-channel binding site on flow is derived. (PDF) [file pone.0015160.s002.pdf]

Appendix S2

Transport of independent particles: We first treat the highly artificial but analytically simplest case of independent, i.e. not interacting, particles. Although these results do not describe correctly the physical situation, the quantities derived will enter the actual solution and these results serve as a reference for the effects of particle-particle interaction.

In the steady state reactive fluxes at the channel ends and diffusive flows are equivalent. For independent particles one obtains for flow from bath A to bath B

$$\left.\begin{array}{l} k_+^{(A)}\, c_A - k_-^{(A)}\, \rho_s(0) \\ -\, D[\partial_x - F(x)]\, \rho_s(x) \\ k_-^{(B)}\, \rho_s(L) - k_+^{(B)}\, c_B \end{array}\right\} \equiv J_0 \tag{S2-1}$$

Instead of solving the last equations directly, we derive steady state flow in terms of occupation numbers and first passage times. For unidirectional transport from bath A to B , $(c_A \neq 0,\; c_B = 0)$ the Fundamental Equation holds [1, 2],

$$J_0 = \frac{N_{0\to B}}{\tau_{0\to B}},\ \text{with}\ \ N_{0\to B} = \int_0^L \rho_s(x)\, dx\ , \tag{S2-2}$$

relating steady state flow to the number of particles in the channel, $N_{0\to B}$, and the regular first passage time, $\tau_{0\to B}$. This equation holds for any unidirectional transport through some domain from some adjacent source to an adjacent absorber, i.e. for any topology, dimension of the latter and for any interaction of particles with each other or with the domain. Regular first passage time means that a reflecting boundary is employed at the source (here $x = 0$) and that the particle is absorbed once it reaches the absorber (here bath B). This is denoted by the subscript $0 \to B$, which also labels the particle number. For independent particles the particle number in the channel is proportional to the bath concentration c_A [2], which suggests the introduction of a specific particle number n, independent of the concentration,

$$n_{0\to B} = N_{0\to B}/c_A\ . \tag{S2-3}$$

A similar relation holds for unidirectional flow from B to A $(c_A = 0, c_B \neq 0)$. For arbitrary concentrations in the baths, total flow is simply the superposition of the unidirectional flows since particles are not interacting with each other,

$$J_0 = \frac{n_{0\to B}}{\tau_{0\to B}}\, c_A - \frac{n_{L\to A}}{\tau_{L\to A}}\, c_B\ \ . \tag{S2-4}$$

A vanishing flow for equal concentrations $c_A = c_B$ implies equivalence of the corresponding diffusive conductivities, $n_{0\to B}/\tau_{0\to B}$ and $n_{L\to A}/\tau_{L\to A}$, which also holds for any linear combinations, i.e.

$$\frac{n_{0\to B}}{\tau_{0\to B}} = \frac{n_{L\to A}}{\tau_{L\to A}} = \frac{\alpha\, n_{0\to B} + \beta\, n_{L\to A}}{\alpha\, \tau_{0\to B} + \beta\, \tau_{L\to A}} \tag{S2-5}$$

For the following it is particularly useful to introduce the symmetrized $(\alpha = \beta = 1/2)$ and antisymmetrized $(\alpha = -\beta = 1/2)$ specific particle number and first passage time:

$$n = \frac{1}{2}\,(n_{0\to B} + n_{L\to A}),\ \ \tau = \frac{1}{2}\,(\tau_{0\to B} + \tau_{L\to A})\ , \tag{S2-6}$$

$$\Delta n = \frac{1}{2}\,(n_{0\to B} - n_{L\to A}),\ \ \Delta\tau = \frac{1}{2}\,(\tau_{0\to B} - \tau_{L\to A})\ . \tag{S2-7}$$

Symmetrized, antisymmetrized, as well as unidirectional quantities have the property

$$\frac{n}{\tau} = \frac{\Delta n}{\Delta\tau} = \frac{n_{0\to B}}{\tau_{0\to B}} = \frac{n_{L\to A}}{\tau_{L\to A}}\ . \tag{S2-8}$$

Flow can now be written in the form of a macroscopic Fick's diffusion law

$$J_0 = \frac{n}{\tau}\,(c_A - c_B)\;. \tag{S2-9}$$

Results for a general channel: We will now determine the symmetrized specific particle number n and the symmetrized first passage time τ explicitly for a general channel.

For determination of the first passage times, we exploit that they are additive for one-dimensional diffusion. For transport from bath A to B

$$\tau_{0\to B} = \tau_{0\to L} + \tau_{L\to B} \tag{S2-10}$$

holds, where

$$\tau_{0\to L} = D^{-1}\int_0^L dx\; e^{\Phi(x)}\int_0^x d\xi\; e^{-\Phi(\xi)} \tag{S2-11}$$

is the regular first passage time of a particle starting at the reflective boundary $x = 0$, which is absorbed at $x = L$. The second term

$$\tau_{L\to B} = L\;\langle e^{-(\Phi(x)-\Phi(L)}\rangle\;\frac{1}{k_-^{(B)}} \tag{S2-12}$$

is the first passage time of a particle starting at $x = L$, which is reflected at $x = 0$, and absorbed in bath B [3], where the brackets denote the spatial average $\langle\;\rangle = L^{-1}\;\int_0^L dx$. Similar relations hold for transport from bath B to A, and the symmetrized and antisymmetrized first passage times result in

$$\begin{aligned} \tau \;&=\; \frac{L^2}{2D}\langle e^{-\Phi(x)}\rangle\langle e^{\Phi(x)}\rangle \\ &+\; \frac{L}{2}\langle e^{-(\Phi(x)}\rangle\left(\frac{e^{\Phi(L)}}{k_-^{(B)}}+\frac{e^{\Phi(0)}}{k_-^{(A)}}\right) \end{aligned} \tag{S2-13}$$

$$\begin{aligned} \Delta\tau \;&=\; \frac{L^2}{2D}\langle\langle \mathrm{sgn}(x-y)\; e^{-[\Phi(x)-\Phi(y)]}\rangle\rangle \\ &+\; \frac{L}{2}\langle e^{-\Phi(x)}\rangle\left(\frac{e^{\Phi(L)}}{k_-^{(B)}}-\frac{e^{\Phi(0)}}{k_-^{(A)}}\right)\;, \end{aligned} \tag{S2-14}$$

with $\langle\langle\;\rangle\rangle := L^{-2}\int_0^L\int_0^L dxdy$ and $\mathrm{sgn}(z)$ being the sign function, i.e. $\mathrm{sgn}(z) \equiv -1,\; z < 0$ and $\mathrm{sgn}(z) \equiv 1,\; z \geq 0$. Note that for fast exchange, $k_- \to \infty$, the last term vanishes in both equations, leaving the well known results of the purely diffusive symmetrized and antisymmetrized first passage times [2].

The specific particle number n is obtained from equilibrium conditions, i.e. when $c_A = c_B = c$. When the baths A and B are on the same free energy level, i.e. $\Phi(0) = \Phi(L)$, the density fulfills $\rho_s(x) = \rho_{eq}(x) = c\;e^{-[g+\Phi(x)-\Phi(0)]}$. The number of particle inside the channel is $N_{eq} = c\int_0^L dx\;e^{-[g+\Phi(x)-\Phi(0)]}$, or written in terms of the the specific particle numbers $N_{eq} = c\;n_{0\to B} + c\;n_{L\to A} = 2\;c\;n$. So the symmetrized specific particle number has the form

$$n = \frac{L}{2}\;\langle e^{-[g+\Phi(x)-\Phi(0)]}\rangle\;. \tag{S2-15}$$

We emphasize that with Eq. (S2-8) the quantities Δn, $n_{0\to B}$, and $n_{L\to A}$, respectively, can be derived from n, τ, and $\Delta\tau$.

Channel occupation probabilities for interacting particles: For interacting particles one has to replace the concentrations in Eqs. (S2-1) by their probability weighted values,

$$(c_A,\;c_B) \to (P_0\;c_A,\;P_0\;c_B)\quad, \tag{S2-16}$$

where P_0 is the probability to find the channel unoccupied. For the determination of P_0, one applies conservation of probability, i.e. the channel is either unoccupied with probability P_0, or occupied with probability P_1, i.e. $P_0 + P_1$=1.

The definition of the specific particle numbers, Eq. (S2-3), and the formal replacement (S2-16) then imply

$$\underbrace{P_0\ c_A\ n_{0\to B} + P_0\ c_B\ n_{L\to A}}_{=P_1} + P_0 = 1\ . \tag{S2-17}$$

Consequently, the probability to find an empty channel is

$$\begin{aligned} P_0 &= \frac{1}{1 + c_A\ n_{0\to B} + c_B\ n_{L\to A}} \\ &= \frac{1}{1 + n(c_A + c_B) + \Delta n(c_A - c_B)}\ . \end{aligned} \tag{S2-18}$$

For the case of m different species, with respective concentrations $c^{(i)}$ and respective specific particle numbers $n^{(i)}_{0\to B}$, $n^{(i)}_{L\to A}$, the considerations concerning conservation of probability also hold,

$$\underbrace{\sum_{i=1}^{m} P_0 c_A^{(i)} n_{0\to B}^{(i)} + P_0 c_B^{(i)} n_{L\to A}^{(i)}}_{=P_1} + P_0 = 1\ , \tag{S2-19}$$

resulting in a generalization of Eq. (S2-18),

$$\begin{aligned} P_0 &= \frac{1}{1 + \sum_{i=1}^{m}\left[c_A^{(i)} n_{0\to B}^{(i)} + c_B^{(i)} n_{L\to A}^{(i)}\right]} \\ &= \frac{1}{1 + \sum_{i=1}^{m}\left[n^{(i)}\ (c_A^{(i)} + c_B^{(i)}) + \Delta n^{(i)}\ (c_A^{(i)} - c_B^{(i)})\right]}\ . \end{aligned} \tag{S2-20}$$

Translocation probability: The translocation probability is the conditional probability that a particle located at one channel end is absorbed by the bath at the opposite end. For a particle at $x = 0$ this conditional probability can be derived from steady state flows of unidirectional transport $A \to B$, ($c_A = c \neq 0,\ c_B = 0$) of independent particles. The number of particles entering per unit time the channel at $x = 0$ from bath A is

$$J_{A\to 0} = k_+^{(A)} c\ . \tag{S2-21}$$

This flow splits up into the flow of particles which either leave the channel by absorption into bath B, given by J_0, or back into bath A. Hence, the translocation probability is the ratio

$$p_{0\to B} = \frac{J_0}{J_{A\to 0}} = \frac{n/\tau}{k_+^{(A)}}\ , \tag{S2-22}$$

i.e. the ratio of the diffusive conductivity to reactive conductivity at the entrance. We note that — within our approximative treatment of particle-particle interaction resulting in channel blocking — this quantity is identical for independent and interacting particles. This relation directly implies that the translocation probability mainly increases with overall binding strength, similar to J_0, and is independent from the detailed arrangement of interactions within the channel, i.e. permutations of $\Phi(x)$.

Lifetime of channel states and occupation probabilities: For simplicity we consider again unidirectional transport ($c_A = c \neq 0,\ c_B = 0$). The mean time for channel access, i.e. the mean lifetime of an empty channel is

$$\tau_e^{(A)} = 1/(k_+^{(A)}\ c)\ . \tag{S2-23}$$

Once the particle has reached the channel entrance, $x = 0$, it stays inside the channel for a certain residence time before it leaves either by returning into bath A or by passing through to bath B. Determining the mean residence time, $\tau_{A\leftarrow 0\to B}$, is well established [3–5]. Here, however, we want to derive it in terms of occupation number and first passage time. For this purpose we apply Eq. (S2-2), which holds for every unidirectional flow when the corresponding first passage time is inserted. We consider now the flow component from bath A to $x = 0$, $J_{A\to 0} = k_+^{(A)} c_A$ for

independent particles. The first of the Eqs. (S2-1) than may be reinterpreted that at $x = 0$ this flow splits up into flow of particles reabsorbed by bath A, and diffusive flow toward bath B, i.e. $k_+^{(A)} c_A = k_-^{(A)} \rho(0) + J_0$. The first passage time of this process is just the mean residence time $\tau_{A\leftarrow 0\rightarrow B}$. The particle number is independent from the flow under consideration, so $N_{A\leftarrow 0\rightarrow B} = N_{0\rightarrow B}$, which according to Eq. (S2-2) results in

$$k_+^{(A)} c_A = \frac{N_{0\rightarrow B}}{\tau_{A\leftarrow 0\rightarrow B}} \tag{S2-24}$$

The Eqs. (S2-3,S2-5) then imply

$$\tau_{A\leftarrow 0\rightarrow B} = \frac{n_{0\rightarrow B}}{k_+^{(A)}} = \frac{n}{k_+^{(A)}} \, \frac{\tau_{0\rightarrow B}}{\tau} \ , \tag{S2-25}$$

which in the case of a symmetric channel ($\tau = \tau_{0\rightarrow B}$, $k_+^{(A)} = k_+^{(B)} = k_+$) simplifies to $\tau_{A\leftarrow 0\rightarrow B} = n/k_+$. Hence, the ratio of lifetimes of empty to occupied channel is

$$\frac{\tau_e^{(A)}}{\tau_{A\leftarrow 0\rightarrow B}} = \frac{n}{\tau} \, \tau_{0\rightarrow B} \; c \ . \tag{S2-26}$$

The same holds for unidirectional transport in the opposite direction., i.e. one obtains for the symmetrized and antisymmetrized ratios of lifetimes of channel states

$$\begin{aligned} \frac{\tau_e^{(A)}}{\tau_{A\leftarrow 0\rightarrow B}} \pm \frac{\tau_e^{(B)}}{\tau_{A\leftarrow L\rightarrow B}} &= \underbrace{\frac{n}{\tau} \; (\tau_{0\rightarrow B} \pm \tau_{L\rightarrow A})}_{=2n \text{ or } 2\Delta n} \; c \\ &= \left(\frac{P_1}{P_0}\right)_{A\rightarrow B} \pm \left(\frac{P_1}{P_0}\right)_{B\rightarrow A} \end{aligned} \tag{S2-27}$$

where we exploited Eqs. (S2-5-S2-7). So the ratios of mean lifetimes of channel states determine ratios of channel occupation probabilities.

Effect of an asymmetric binding site on flow: As a generic model for a binding site we assume a rectangular shaped potential $\Phi(x)$, which has a depth $\Phi_0 < 0$ within the binding region of width wL ($0 < w \leq 1$), and is otherwise zero. Asymmetry is realized by shifting the center of the binding site a distance κL away from the middle of the channel $L/2$. To keep the binding site within the channel, the asymmetry parameter must fulfill $|\kappa| \leq (1 - w)/2$. Insertion into Eqs. (S2-13, S2-14, S2-15) gives symmetrized and antisymmetrized first passage times and specific particle numbers as

$$\begin{aligned} \tau &= \frac{L^2}{2D} \left(1 + 4w(1-w)\sinh^2(\Phi_0/2)\right) \\ &+ \frac{L}{2}(1 + w(e^{-\Phi_0} - 1)) \left(\frac{1}{k_-^{(B)}} + \frac{1}{k_-^{(A)}}\right) \end{aligned} \tag{S2-28}$$

$$\begin{aligned} \Delta\tau &= \underbrace{\frac{L^2}{2D} \, 4\kappa w \, \sinh(\Phi_0)}_{=(\tau_{0\rightarrow L} - \tau_{L\rightarrow 0})/2, \text{ asymmetry of } \Phi(x)} \\ &+ \underbrace{\frac{L}{2}(1 + w(e^{-\Phi_0} - 1)) \left(\frac{1}{k_-^{(B)}} - \frac{1}{k_-^{(A)}}\right)}_{=(\tau_{L\rightarrow B} - \tau_{0\rightarrow A})/2 \text{ , asymmetry of channel exit}} \end{aligned} \tag{S2-29}$$

$$n = \frac{L}{2} \, e^{-g} \, \left(1 + w(e^{-\Phi_0} - 1)\right) \ . \tag{S2-30}$$

The parameter Δn, which is necessary for determining the probability to find the channel either empty P_0 (see

Eq. (S2-18), or occupied $P_1 = 1 - P_0$, is obtained from Eq. (S2-8) as

$$\Delta n = \frac{n}{\tau}\,\Delta\tau\;. \tag{S2-31}$$

The symmetrized parameters τ and n, and, hence, n/τ, are independent of permutations of the potential $\Phi(x)$, i.e. in particular of location of the binding site, quantified by the parameter κ. The antisymmetrized first passage time $\Delta\tau$ consists of two components, namely the part related to diffusion within the asymmetric potential $\Phi(x)$ and the part related to the difference of first passage times of channel exit. The latter depends on exit rates and the averaged Boltzmann factor $\langle e^{-\Phi(x)}\rangle = 1 + w(e^{-\Phi_0} - 1)$ (Eq. (S2-12)),i.e. it is independent of permutations of $\Phi(x)$, in particular of the location of the binding site. So the effect of the asymmetry of $\Phi(x)$, and of the asymmetry of channel exit can be discussed separately.

First, symmetric exchange properties at the channel ends are assumed, which let the term related to channel exit in Eq. (S2-29) vanish. A binding site near bath B ($\kappa > 0$) traps particles in this location. For unidirectional transport $A \to B$ this trapping in trans position, i.e. close to the absorbing bath, enhances the rate of the particles reaching the channel end $x = L$, when compared to the rate of particles reaching the channel end $x = 0$ for unidirectional flow in reverse direction $B \to A$. This is reflected in the corresponding first passage times, i.e. $\Delta\tau = (\tau_{0\to B} - \tau_{L\to A})/2 = (\tau_{0\to L} - \tau_{L\to 0})/2 < 0$. Intuitively it is evident, that this asymmetry of first passage times is accompanied by an asymmetry in occupation probabilities. For unidirectional transport $A \to B$, the corresponding shorter first passage time should imply a higher probability to find the channel empty, than for unidirectional transport in reverse direction $B \to A$. Mathematically this can be seen from Eq. (S2-31), as $\Delta\tau < 0$ implies $\Delta n < 0$ (note that n/τ in is independent of κ). When inserted into Eq. (S2-18), one obtains the result anticipated intuitively,

$$\begin{aligned}\Delta P_0 &= \underbrace{P_0(c_A = c, c_B = 0)}_{\text{unidirect. transport } A\to B} - \underbrace{P_0(c_A = 0, c_B = c)}_{\text{unidirect. transport } B\to A} \\ &= -2\Delta n\; P_0(c,0)\; P_0(0,c) > 0\;.\end{aligned} \tag{S2-32}$$

The difference of unidirectional flows is then

$$\Delta J = \Delta P_0\; J_0 > 0\;, \tag{S2-33}$$

as J_0 is independent from asymmetry. Hence, a binding site in trans position of the concentration gradient implies a higher flow than in cis position.

We now consider a symmetric particle-channel interaction $\Phi(x)$, but assume asymmetric exchange at the channel ends, i.e. the exit rates differ. Hence the antisymmetrized first passage time, Eq. (S2-29), solely consists of the term related to channel exit. If channel exit to bath B is faster than to bath A, i.e. $k_-^{(B)} > k_-^{(A)}$, the first passage time for transport $A \to B$ is shorter than that in reverse direction, $B \to A$, i.e. $\Delta\tau < 0$. Following the same arguments as above, one obtains a higher probability to find the channel empty, and by this a higher flow for unidirectional transport from $A \to B$, than for $B \to A$. In the general case when an asymmetric binding site and channel exit are present, the Eqs. (S2-29,S2-31) imply that both components may work synergistically, i.e. may enhance flow asymmetry, when the binding site is close to the end with the faster exit rate k_- and vice versa they may counteract when binding is located near the end with the slower exit rate.

[1] Hardt S (1981) Diffusion transit time - a simple derivation. *Bull Math Biol*, 43, 89-99

[2] Bauer WR, Nadler W (2005) Stationary flow, first passage time, and macroscopic Fick's first diffusion law: application to flow enhancement by particle trapping. *J Chem Phys*, 122, 244904

[3] Schulten K , Schulten Z, Szabo A (1981) Dynamics of reactions involving diffusive barrier crossing. *J Chem Phys*, 74, 4426-4432

[4] Szabo A, Schulten K, Schulten Z (1980) First passage time approach to diffusion controlled reactions. *J Chem Phys*, 72, 4350-4357

[5] Berezhkovskii AM, Pustovolt MA, Bezrukov SM (2003) Channel-facilitated membrane transport: Average lifetime in the channel. *J Chem Phys*, 119: 3943-3951
