# Supplementary material for: Thermodynamics of Competitive Molecular Channel Transport: Application to Artificial Nuclear Pores
Source: PLoS One. 2010 Dec 13;5(12):e15160. doi: 10.1371/journal.pone.0015160 (PMC3001458; doi:10.1371/journal.pone.0015160)
Supplement: Appendix S3 — The access dynamics of the nuclear transport factor (NTF) from outside to the nuclear pore is estimated. Furthermore the diffusive conductivities of bovine serum albumin (BSA) and nuclear transport factor (NTF) through the nuclear pores and their activities are computed from experimental data. (PDF) [file pone.0015160.s003.pdf]

Appendix S3

Estimation of access dynamics for nuclear pores: This follows from the Smoluchowski Equation, which quantifies entry into the pore by the absorption rate of a perfectly absorbing disk (radius r) representing the orifice of the pore (diameter$\approx$ 30nm) , $k_+ = 4rD_{\text{in solvent}}$ [1], where $D_{\text{in solvent}}$ is the diffusion coefficient in the solvent, which for BSA or nuclear transport factors was taken to be 6.8 10^{-11} m^2/s. Since concentrations c in the experiments were in the μM range, the mean time that a channel stays empty is $\tau_e = 1/(k_+\ c) \approx 0.4$ms. Flow in this range of concentrations had a magnitude of 1 molecule/(pore$\times$ s), i.e. three orders of magnitude slower than channel access.

Diffusive conductivities of BSA and NTF: In their experiments Jovanovic-Talismann et al. determined ratios of fluxes through functionalized (NSP1, 30 kDa PEG-thiol) pores related to non-functionalized control pores. For pores functionalized with NSP1 they obtained a four times higher flux ratio for NTF than for BSA, i.e.

$$[J^{(\text{NTF})}_{\text{NSP1}}/J^{(\text{NTF})}_{\text{control}}]/[J^{(\text{BSA})}_{\text{NSP1}}/J^{(\text{BSA})}_{\text{control}}] \approx 4\ . \tag{S3-1}$$

Respecting that $J = P_0 J_0$ for any species and pore, and that P_0 of NTF and BSA is identical for each pore, i.e. it cancels out in flow ratios, one obtains

$$\frac{J^{(\text{NTF})}_{\text{NSP1}}/J^{(\text{NTF})}_{\text{control}}}{J^{(\text{BSA})}_{\text{NSP1}}/J^{(\text{BSA})}_{\text{control}}} = \frac{J^{(\text{NTF})}_{\text{NSP1}}}{J^{(\text{BSA})}_{\text{NSP1}}} \times \frac{J^{(\text{BSA})}_{0,\text{control}}}{J^{(\text{NTF})}_{0,\text{control}}}\ . \tag{S3-2}$$

In the control pore BSA and NTF should behave similar concerning their transport properties, i.e. $(n/\tau)^{(\text{BSA})}_{\text{control}} = (n/\tau)^{(\text{NTF})}_{\text{control}}$. Hence, one has $[J^{(\text{BSA})}_{0,\text{control}}/J^{(\text{NTF})}_{0,\text{control}}] = c_{\text{BSA}}/c_{\text{NTF}}$, and finally for the ratio of NTF and BSA conductivities in the NSP1 functionalized pore

$$\frac{J^{(\text{NTF})}_{\text{NSP1}}/c^{(\text{NTF})}}{J^{(\text{BSA})}_{\text{NSP1}}/c^{(\text{BSA})}} = 4\ . \tag{S3-3}$$

Determination of activities of BSA and NTF: For determination of the activities Lce^{-g} we inserted the concentrations 1.5 μM for BSA and 0.75μM for NTF, respectively, as given by Jovanovic-Talismann et al.. Since there is no evidence for a significant energetic or entropic barrier a nuclear transport factor or BSA has to overcome to enter the pore, we assume no relevant energetic difference for the reaction at the pore ends. Hence, the standard free energy must solely match the 3-D concentration to the one-dimensional concentration in the pore, i.e. $e^{-g} = \pi\ r^2$, with $r = 15$nm as the pore radius (pore diameter 30 nm). The length was taken to be 6μm. With the Loschmidt constant one derives then activities as $Lce^{-g} = 2.55 \times$ concentration in μM.

---

[1] Berg H (1983) in *Random Walks in Biology*, (Princeton University Press, Princeton, NY)
